# Retraction: Myocardial Metabolism and Postoperative Risk Stratification in Cardiac Surgery: A Narrative Review and Conceptual Framework

**DOI:** 10.7759/cureus.r238

**Published:** 2026-08-26

**Authors:** John Salib, Mark Salib, Murali K Manikkavelu, Frederick Tiesenga

**Affiliations:** 1 School of Medicine, St. George's University, St. George's, GRD; 2 General Surgery, Community First Medical Center, Chicago, USA; 3 General Surgery, American University of Antigua, St. John's, ATG; 4 General Surgery, West Suburban Medical Center, Chicago, USA

The Editor-in-Chief has retracted this article. Following a reader complaint, the editorial office conducted a post-publication assessment of the article's citations. It was determined that 33 of the 44 references do not support the corresponding cited article text.

The authors were queried on each item and responded. They confirmed all of the findings, and proposed corrections. However, the extent of the required revision, which affects the majority of the article's citations, the composition of the reference list, the reported search accounting, and the evidentiary basis of several of the article's claims, exceeds what can be appropriately addressed via correction. As a result, this article has been retracted in order to preserve the scientific record.

The authors agree with the decision to retract.

